# Clinical, epidemiological, and therapeutic profile of patients with a retinoblastoma diagnosis experience in the Costa Rica National Children’s Hospital Oncology Unit from January 2009 to December 2015

**DOI:** 10.3332/ecancer.2017.754

**Published:** 2017-07-24

**Authors:** Jennie Chen Lo, Carlos Rodríguez, Rigoberto Monestel, Arnoldo Zúñiga

**Affiliations:** 1 Radiation Therapy Unit, Department of Hematology-Oncology, Mexico Hospital, CCSS, San José, Costa Rica; 2 Oncology Unit, Department of Hematology-Oncology, National Children’s Hospital, CCSS, San José, Costa Rica

**Keywords:** retinoblastoma, radiation therapy, response to treatment

## Abstract

**Introduction:**

Retinoblastoma represents only 3% of paediatric cancers, but it is the most prevalent intraocular tumour in this population. It develops in the retina as a primitive neuroectodermal tumour that affects development during gestation. The tumour presents in two different forms depending on whether or not it expresses a genetic modification. For patients diagnosed at preschool age, 75% are unilateral non-hereditary cases. While enucleation is the preferred treatment for advanced stages of the tumour, other modalities, such as systemic and intraocular chemotherapy, radiotherapy and local treatments with thermotherapy, cryotherapy, and brachytherapy can be used to try to preserve the eye. However, applying radiation therapy treatments increases the risk of secondary tumours.

**Objective:**

To analyse the results obtained from patients with a retinoblastoma diagnosis at the Costa Rica National Children’s Hospital (HNN) Oncology Unit who received external beam radiation therapy and other therapeutic modalities during the period from January 2009 to December 2015.

**Materials and methods:**

Data were extracted from the patient’s medical records and entered in a data collection instrument. We then analysed the results and formulated conclusions.

**Results:**

A total of 36 patients were evaluated. This corresponded to 45 cases or the number of eyes affected by bilateral presentation of retinoblastoma. The documented incidence was 0.83 cases per 10,000 live births and the majority were female preschool-age children. Of these, 40% presented bilaterally and 13% were of hereditary origin. Up to 78% of these cases were diagnosed with advanced stage D and stage E. All patients who received conservative treatment progressed, requiring up to four lines of treatment. Eight patients, all in the most advanced stage, received external beam radiation due to the failure of other modalities. The main adverse effects observed were radiodermatitis, facial hypoplasia and conjunctivitis. Additionally, we report the emergence of a secondary neoplasm in two patients, one post-chemotherapy and the other post-radiotherapy.

**Conclusions:**

Advanced-stage patients who initially received conservative treatments responded more poorly than those treated more aggressively with surgery alone or with surgery combined with another treatment modality. Treatment with radiation therapy was used in 22% of the cases (8 patients) and all patients treated with radiotherapy showed some adverse effects.

## Introduction

Retinoblastoma represents only 3% of paediatric cancers, but it is the most prevalent intraocular tumour in this population. The variation in global incidence is attributed to the genetic susceptibility and environmental exposure of each patient. About 3000 children die from retinoblastoma every year in the world, with the highest incidence in Asia and Africa [1–4].

It develops in the retina as a primitive neuroectodermal tumour associated with some event that affects development during gestation [1–3, 5].

There are two different forms of tumour according to its genetic involvement. It is associated with different clinical presentations mainly on laterality and mechanism of tumour development; however, both have a similar histology [1–3].

Usually, patients are diagnosed at preschool age, and 75% of cases are non-heritable and unilateral. Predictive factors for this disease that should be taken into account are advanced disease, extraocular, metastatic and trilateral retinoblastoma, which refers to the presence of a bilateral disease associated with a pineoblastoma [2, 3, 6].

Retinoblastoma is potentially curable, as its prognosis has improved considerably in recent decades. Enucleation is still the mainstay of treatment for advanced stage tumours; while conservative modalities, seeking to salvage the eyeball with the proper preservation of vision, include systemic intraocular and intra-arterial chemotherapy, radiotherapy and local treatments with thermotherapy, cryotherapy, and brachytherapy [2, 3, 6–10].

The emergence of second malignant neoplasms is mainly associated with germinal or hereditary retinoblastoma patients. Applying treatments with radiotherapy further increases the possibility that these will arise; therefore, there is a tendency to radiate less and use more treatment with chemotherapy [2, 3, 6].

External beam radiotherapy has been considered one of the pillars in the treatment of retinoblastoma, conservatively in patients with early disease with a good vision level and in the adjuvant in cases of advanced diagnosis [2, 4, 6].

The purpose of this study is to perform a clinical, epidemiological, and response characterisation of different treatment modalities for patients with a diagnosis of retinoblastoma at the Costa Rica National Children’s Hospital (HNN) Oncology Unit for a set period of time.

This study intends to publicise local experience, as there are currently very few prior studies related to this topic in our country.

### General objective

To analyse the results obtained from patients with a diagnosis of retinoblastoma in the Costa Rica National Children’s Hospital Oncology Unit who received external beam radiation therapy and other therapeutic modalities during the period from January 2009 to December 2015.

### Specific objectives

- To describe the incidence of retinoblastoma by sex and age of the population under study.
- To identify the indications taken into account for the choice of treatment (surgical, medical, and/or radiotherapy) by stage of the disease.
- To describe the radiotherapy regimen used (technique, number of fields, doses received).
- To list the main adverse effects shown during and after treatment with radiotherapy.

## Materials and methods

This study is descriptive and observational and covers a series of cross-sectional and retrospective cases.

The study describes the incidence, characteristics, and responses to the different treatment modalities of a disease in a given population and documents the association between two or more variables without assuming a causal relationship between them.

This study describes the clinical and epidemiological characteristics of paediatric patients with a diagnosis of retinoblastoma in the Pediatric Oncology Unit of the National Children’s Hospital of the Costa Rican Department of Social Security between January 2009 and December 2015.

The characteristics selected in the predetermined population are listed in the series of cross-sectional cases. In addition, this is considered to be a retrospective study, as it was designed and completed subsequent to the facts studied. Data were obtained from information recorded in electronic and physical clinical records, and these data were entered into the collection instrument, which in turn permitted the analysis and measurement of the study variables. Inclusion criteria were age less than 18 years for both genders with a diagnosis of retinoblastoma, treated between January 2009 and December 2015. Exclusion criteria were applied to patients with incomplete records. This was defined as the absence of data from more than 50% of the study variables. In addition, those patients who did not complete treatment were excluded.

We used the data collection sheet, which was a list of questions about the characteristics related to the different variables; all these data were collected in written form by the same researcher by reviewing the digital or physical clinical records of patients who met the criteria for inclusion in the study. After data collection, the management and analysis of the information were carried out electronically.

Statistical techniques used in the analysis of the information from the descriptive analysis of the population include: simple frequency tables, association or contingency tables, and descriptive statistics for average, minimum, maximum, standard deviation, quartiles, and incidence rates by age groups and years of study. For survival analysis, Kaplan–Meier survival curves and incidence rates of death per person-time were used. The software used to analyse the data was Stata 14.

This study was approved by the Scientific Ethics Committee of the Costa Rica National Children’s Hospital on 13 January 2016, with the assigned protocol number CLOBI-HNN-006-2016.

## Results

A total of 36 patients were evaluated. This corresponded to 45 cases of eyes affected by bilateral presentation of retinoblastoma. The incidence of this disease in Costa Rica in the study period is higher in children under one year compared with other age groups that present this disease. In this age group, the rate has been maintained over the last seven years between 0.14 and 0.28 per 10,000 people, while in the 1–3-year-old group, there were marked declines with two peaks in 2010 and 2013, but with lower values of the rate of incidence of those under one-year old. These data were calculated according to population-based registries from the National Institute of Statistics and Census of Costa Rica (Table 1).

The results obtained showed that this was more common in females, and 55.6% of the population studied was female.

The average age of the patients at the time of retinoblastoma diagnosis was 1.6 ± 1.6 years. The youngest was 0.08 (1 month) and the oldest 7.0 years, with 50% of the patients aged between 0.8 years (9.6 months) and 2.0 years. It should be noted that there were three extreme values: two male patients aged 5.0 and 7.0 and a female aged 7.0 (Table 2).

It has been documented that 25% (9 patients) of the study population had a bilateral presentation.

13.3% of the cases studied (affected eyes) were classified as hereditary/genetic, 86.7% fall in the category of sporadic retinoblastoma confirmed by fluorescent *in situ* hybridisation for the RB1 gene.

**Figure 1.**
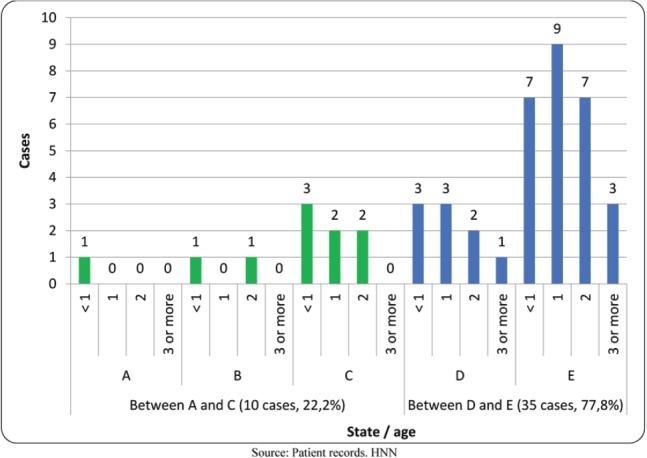
Stage of retinoblastoma patients in study by age. HNN. 2009 to 2015. (*N* = 45 cases). Source: patient records, HNN.

The International Classification of Retinoblastoma stages that occurred most frequently were advanced, with 77.8% of cases presenting in stages D and E, stage E being the most common. Only three cases (6.6%) of the total studied were classified in the early stages with better prognosis, A and B. None of the patients presented extraocular or metastatic disease (Figure 1).

## Treatments administered

It is considered that the most appropriate treatment for retinoblastoma must take into account both age and stage at diagnosis. Cases of early stages (A and B) can be treated with local therapy only, or associated with systemic chemotherapy. Only if progression is documented should the use of external radiotherapy be considered. For patients at stage C and D, the aforementioned treatments are also used; however, if massive vitreous seeding is detected after chemotherapy, adjuvant therapy with external radiotherapy should be considered. All these treatment modalities are considered to be conservative management, as they seek to salvage the affected eye. For patients at stage E, the primary treatment recommended is radical management with surgery with enucleation of the affected eye. All treatment that includes surgery with enucleation, with or without other treatment modalities, is considered to be aggressive treatment [11–13].

In this study, we identified that after applying the first line of treatment to patients, the following responses were observed. 57.8% of cases received a complete response. Nine of these cases received surgery only, while 16 of them were given a combination of surgery with systemic chemotherapy; all of these were considered to be aggressive management. Only one patient who received conservative treatment with cryotherapy, systemic chemotherapy and radiotherapy achieved a complete response. 96.1% of these cases were at stages D to E of the disease; 61.5% of the cases also received intravenous chemotherapy. Only 7.7% of the cases with a complete response were between stages A and C of the disease.

All cases with progressive disease (28.9%, 13 cases) received conservative treatment, except in one of the cases where this was combined with surgery, but the patient died due to progression despite aggressive management. In this case and in seven other cases, the disease was at late stages between D and E, and the others were in early stages from A to C.

Only six (13.3%) of the total number of cases reported stable disease after treatment; four cases were at an early stage and two at a late stage. All cases were treated with conservative modalities.

As for the response obtained in those cases that received a second line of treatment following progression of their disease after the first line of treatment (12 cases), only the enucleated (16.7%) presented a complete response to treatment, with one early stage and one late. Some 66.7% saw a progression of their illness. All of these patients received conservative treatments, and of these, five were treated at a late stage and three at an early stage. The remaining 16.7%, one at an early stage and another at a late stage, were also conservatively treated and showed stable disease.

Specifically, in the cases treated with external radiotherapy, one showed stable disease and two showed progression following treatment.

The third line of treatment after a new progression showed the following results: three (37.5%) patients who underwent surgery obtained a complete response, one at an early stage and two late, while those treated with conservative methods (50%) showed progression of the disease, all had late-stage or stable disease, and the last was a case who underwent radiotherapy plus cryotherapy at an early stage.

Two of the three cases received a fourth line of treatment. When further progression occurred, they obtained a complete response with surgery, while the third patient treated with brachytherapy achieved stable disease. These three cases were all documented at a late stage (Table 3).

The six cases at stage D who showed a complete response were those who had surgery or surgery plus systemic chemotherapy as the means of treatment, the remaining three cases at stage D who received only systemic chemotherapy showed progression of the disease.

A total of 26 cases studied were diagnosed at the most advanced stage (E), 76.9% (20) received a radical treatment with surgery alone or in conjunction with any other modality of treatment, only one of these did not present a complete response.

The six remaining cases that did not receive surgery as a treatment option showed progression of the disease [4] or stable disease [2].

## Radiotherapy

External radiotherapy is considered one of the pillars in the conservative treatment of retinoblastoma, with sufficient local control and preservation of adjacent organs. In this study, we assessed eight patients who were treated with radiotherapy, and these were 22.2% of the total number of patients [5, 14, 15].

None of the patients who received radiotherapy was classified at stage A. Only one patient was classified at each of stages B and C, while the vast majority of patients, 75% of them, were at an advanced stage D or stage E.

The indications for this treatment mode were failure of local therapy with other treatment modalities in six patients, while the remaining two had tumours near the optic nerve or macula. None of the patients presented extraocular disease.

The total dose of radiation received by all patients was 45 Gy, of which 62.5% received 1.8 Gy per fraction, while the remaining 37.5% received 1.5 Gy per fraction.

For the majority of the patients who received radiotherapy, this was done using multiple treatment fields, from 2 to 5 fields. Only 25% of the cases, corresponding to two cases, used a single field of treatment.

All patients receiving external beam radiation had some adverse effect. The most frequently observed was G1 radiation dermatitis in 62% of the cases, and the second most common were conjunctivitis and facial hypoplasia, both of which occurred in 50% of the cases. The least common side effect was G2 radiation dermatitis, which occurred in only one patient [16].

## Follow-up

The follow-up period of eye examination under anaesthesia of patients with retinoblastoma was initiated after treatment of the last detected active disease in order to determine patient survival as well as the occurrence of secondary tumours after treatment.

Three patients were found to have secondary tumours after treatment, where one presented a histology of right orbital neuroblastoma 1 year after the completion of treatment. This was considered secondary to the systemic chemotherapy received, as it was a patient who did not receive external beam radiation.

The second patient with a secondary tumour presented an embryonic right parietal rhabdomyosarcoma 3 years and 4 months after radiotherapy. Finally, the third patient presented a cavernous hemangioma 4 years and 10 months after the end of treatment, and this was considered of uncertain cause as it received both treatment modalities, external beam radiotherapy and chemotherapy (Table 4).

One death occurred during this study, which occurred during treatment with surgery, systemic chemotherapy, and radiotherapy.

**Figure 2.**
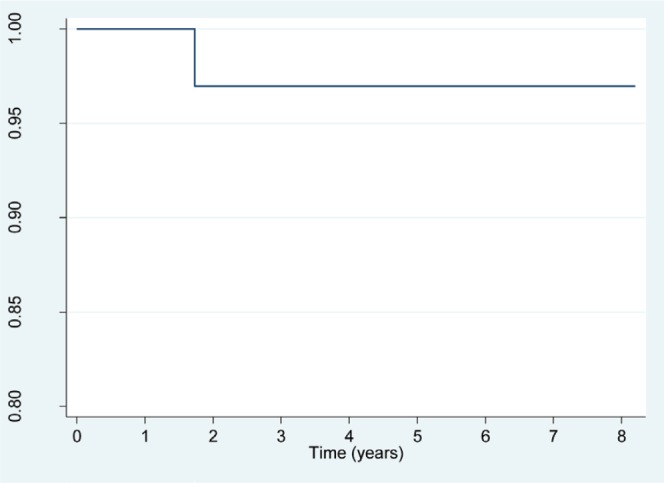
Costa Rica: Survival function for patients with retinoblastoma, from date of diagnosis to study completion date (31 December 2015)—HNN, 2009–2015 (*N* = 36 patients). Source: patient records, HNN.

Use of the Kaplan–Meier estimator for the function of survival between the date of diagnosis and the end of the study showed that the chances of survival after diagnosis are between the 100.00th percentile and the 96.97th percentile, which means that the probability of survival following diagnosis is very high for all National Children’s Hospital patients with retinoblastoma (Figure 2). The death rate for this population was 6.1 per 1000 persons per year.

1: In order to obtain a complete response, patients who received conservative treatments had to receive subsequent aggressive treatment (Table 3).

## Discussion

The incidence of retinoblastoma in Costa Rica during the period 2009–2014 was 0.14 cases per 10,000 people under the age of 1 year and 0.28 per 10,000 people aged 1–3 years. This result is very similar to what is shown in the literature in developed countries such as the United States.

The study population included slightly more women than men, a ratio of 1.3:1. Age at diagnosis was consistent with data in the international literature, with diagnosis most common in preschool children, ages three and under.

The percentage of inherited cases including bilateral, unilateral, or family (13.3%), was lower compared to that reported in the literature (25%). The discrepancy likely resulted from the challenges of performing more precise genetic testing in Costa Rica (this study used fluorescent in situ hybridisation).

Response in advanced-stage patients who first received conservative treatment was worse than in patients treated more aggressively. Those patients either surgery alone or had surgery and received another treatment. Radiotherapy has been crucial in treating retinoblastoma, but only 22.2% of patients in this study received radiation treatment. This figure is consistent with the global trend to use radiotherapy less frequently because it poses a risk of causing second malignant tumours.

Radiotherapy in retinoblastoma patients has a low success rate (12%) and that success did not result exclusively from radiotherapy. The only patient who experienced a complete response had received a combination of systemic chemotherapy, radiotherapy, and surgery.

All patients who received radiotherapy experienced some adverse effects, and 8.3% of all study patients developed a second malignant tumour after treatment. These tumours developed after chemotherapy, radiotherapy or a combination of both treatments.

Patient survival during the study period ranged from the 96.97th and 100th percentiles, similar to the data recorded in developed countries.

## Conclusions

Patients treated with conservative treatment modalities achieve a good outcome, such as stable disease or complete response, when treated in the early stages between A and C. However, patients in the later stages, D and E, present progressive disease if they receive conservative management. Consequently, these patients require aggressive treatment, such as surgical enucleation of the affected eye, in order to achieve a complete response.

All patients who receive a retinoblastoma diagnosis and their families require a multidisciplinary approach. Clinicians and patients need to consider aggressive or radical treatments with surgery for patients with an advanced-stage or late diagnosis of retinoblastoma.

It is important to improve genetic testing techniques in Costa Rica that can identify hereditary cases of retinoblastoma. Physicians also need to provide appropriate genetic counselling to family members of patients.

## Ethical obligations

The investigators have at all times adhered to bioethical principles and are responsible for all information obtained.

The Scientific Ethics Committee of the National Children’s Hospital approved this study.

## Conflicts of interest

The authors declare that they have no conflicts of interest.

## Funding

This study received no funding.

## Figures and Tables

**Table 1.** Costa Rica: Incidence rate of retinoblastoma per 10,000 persons by age group—HNN, 2009–2015 (*N* = 36 patients).

Year	Births	Population 1–3 years	Population 5–7 years	New cases of retinoblastoma			Incidence rate ^*^ 10,000 people		
				< 1 year	1–3 years	5–7 years	< 1 year	1–3 years	5–7 years
2009	75,000	215,522	219,882	2	5	0	0.27	0.23	0.00
2010	70,922	219,906	216,542	1	6	1	0.14	0.27	0.05
2011	73,459	222,845	217,659	2	2	0	0.27	0.09	0.00
2012	73,326	218,840	216,597	1	1	1	0.14	0.05	0.05
2013	70,550	216,934	219,039	1	4	0	0.14	0.18	0.00
2014	71,793	217,854	222,967	2	3	0	0.28	0.14	0.00
2015	71,819	222,299	225,235	2	1	1	0.28	0.04	0.04

Source: patient records, HNN, National Statistics and Census Institute, Information Center.

**Table 2.** Costa Rica: Descriptive statistics of age (years) at the time of diagnosis for patients with a diagnosis of retinoblastoma by gender—HNN, 2009–2015 (*N* = 36 patients).

Gender	Patients	Average	Standard deviation	Minimum	Maximum	Mode	Quartile 1	Quartile 2	Quartile 3
**Total**	36	1.6	1.6	0.08	7.0	1.0	0.8	1.0	2.0
**Female**	20	1.8	1.6	0.08	7.0	2.0	0.9	1.5	2.0
**Male**	16	1.4	1.6	0.25	7.0	1.0	0.6	1.0	1.7

Source: patient records, HNN.

**Table 3.** Costa Rica: Absolute and relative distribution of response by the line of treatment, by the type of treatment and stage of disease. Patients with retinoblastoma—HNN, 2009–2015.

Response to the first line of treatment	Type of treatment	Stage	Cases	Percentage
Stable disease	Conservative	A–C	4	8.8
		D–E	1	2.2
Progressive disease	Aggressive	A–C	0	0
		D–E	1	2.2
	Conservative	A–C	7	15.5
		D–E	5	11.1
Complete response^1/^	Aggressive	A–C	1	2.2
		D–E	26	57.7
***Subtotal***			***45***	***100.0***
**Response to the second line of treatment**	**Type of treatment**	**Stage**	**Cases**	**Percentage**
Stable disease	Conservative	A–C	1	8.3
		D–E	1	8.3
Progressive disease	Conservative	A–C	2	16.6
		D–E	6	50.0
Complete response	Aggressive	A–C	1	8.3
		D–E	1	8.3
***Subtotal***			***12***	***100.0***
**Response to the third line of treatment**	**Type of treatment**	**Stage**	**Cases**	**Percentage**
Stable disease	Conservative	A–C	1	12.5
Progressive disease	Aggressive	D–E	1	12.5
	Conservative	A–C	1	12.5
		D–E	2	25.0
Complete response		D–E	3	37.5
***Subtotal***			***8***	***100.0***
**Response to the fourth line of treatment**	**Type of treatment**	**Stage**	**Cases**	**Percentage**
Stable disease	Conservative	D–E	1	33.3
Complete response	Aggressive	D–E	2	66.7
***Subtotal***			***3***	***100.0***

Source: patient records, HNN.

**Table 4.** Costa Rica: Distribution of retinoblastoma patients who had second tumours after treatment—HNN, 2009–2015.

	Post-chemotherapy	Post-radiation therapy	Uncertain
**Number of cases**	1	1	1
**Histology**	Right orbital neuroblastoma	Right parietal embryonal rhabdomyosarcoma	Cavernous hemangioma
**Time elapsed**	1 Year	3 years and 4 months	4 years and 10 months

Source: patient records, HNN.
